# Supplementary material for: Data integration between clinical research and patient care: A framework for context-depending data sharing and in silico predictions
Source: PLOS Digit Health. 2023 May 15;2(5):e0000140. doi: 10.1371/journal.pdig.0000140 (PMC10184916; doi:10.1371/journal.pdig.0000140)
Supplement: Attachment — Submitted filename: Response_to_reviewers.pdf [file pdig.0000140.s002.pdf]

To the editor and reviewers

Dear Prof. Hinske,

We highly appreciate your as well as the reviewer's insightful and helpful comments on our manuscript. Also, we thank you for giving us the opportunity to further improve the manuscript. We are convinced that we can sufficiently address all the issues raised by you and the reviewers. In addition to the specifically requested issues, we now point out more clearly in the discussion section the differences between this manuscript and our previous work.

Below we provide the point-by-point response to the reviewer's comments. All modifications in the manuscript have been highlighted in red. Upon several requests of the reviewers, we partly reordered and amended the discussion section, while only the novel parts are highlighted.

Sincerely,

Katja Hoffmann

Comments from Reviewer 1:

Thank you for the possibility to review this manuscript. The manuscript provides an interesting description of a software solution that integrates health data and computational analytics (e.g., model predictions, statistical evaluations, visualizations) into a clinical software solution which simultaneously supports both patient-specific healthcare decisions and research efforts. A link to the demonstrator is provided which is very helpful to the reader audience.

Abstract

- Insights from research transferred into clinical routine implemented into clinical practice.
- Software solution: it is much more than software, it is data standardisation, curation, analyses and implementation and you need (software) tools to support this.
- To truly implement insight from research you need tools integrated with / within the **EHR/CIS**. I would expect some reference to this, also in the abstract.

We thank the reviewer for evaluating our manuscript and for the positive feedback. We agree that integration within a CIS or EHR will be necessary for a usable clinical decision support system. However, the implementation of the interface depends on the particular CIS/EHR and on the particular use case. Therefore, further projects will need to determine the specific requirements and select appropriate methods for implementing the integration. To demonstrate how a potential integration could be implemented, we have developed a REST API to provide all model predictions for a requested patient in their respective clinical treatment context within a CIS/EHR. We have referred to this in the abstract (line 42ff), the results (line 343ff) and in the discussion section (line 436ff).

Introduction

- Please write CML in full the first time you use this abbreviation in the full text.

Thank you for the remark. We now explain the abbreviation "CML" when using it for the first time in the Author summary (line 56) and once again in the introduction (line 138).

- It is not clear to me what you mean by "residual disease levels".

We thank the reviewer for this question. "Residual disease levels" commonly refers to a small number of leukemic cells that remain under or after anti-leukemia treatment. Monitoring of this

cell population is an indicator of initial therapy response and also an indicator of possible leukemia recurrence. In order to remove any ambiguity, we rephrased the statement and now refer to “time course data for chronic myeloid leukaemia (CML) patients under ongoing therapy” (line 138).

- Have you considered to compare your framework with general frameworks such as CRISP-DM or Gartner’s AI maturity model?

We thank the reviewer for the reference to the procedural frameworks mentioned, which we were not aware of until now.

Our main focus was on integrating already existing and evaluated computer models in clinical routine processes to automatically calculate individual predictions that can be used to support clinical decisions. The development process of the particular computer models themselves was not the scope of this work. However, we agree, that in Data Mining and AI projects, it is necessary to implement standardized processes to gain an understanding of the medical question and the medical data, for data preparation, modeling, evaluation, deployment and continuous improvement of the models. Therefore, future research should investigate how our software framework could be extended to further support the development process of computer models in clinical research. We have added this interesting aspect to the discussion of the manuscript (line 441ff).

- Next to the mentioned 6 requirements I miss the continuous monitoring of the fit of the model in a changing clinical practice.

We thank the reviewer for the note. In the “Materials and Methods” section of the manuscript, we amended the requirement of the continuous provision of model predictions and analyses in case of changes in the underlying data (treatment, observation, …) (line 150ff).

- The introduction is relatively long and includes both background and problem description as well as some theoretical framework on which the result is base. I would suggest to split this part into an introduction and a method section (which is position after the results section). Please end the introduction with a clear description of the aim of this study. In the method section you can somewhat better clarify the choices of your theoretical framework (which choices made by who and for what reason).

We thank the reviewer for the suggestions, which we have implemented. Specifically, we have divided the introductory section into “Introduction” and “Materials and Methods”. We end the introduction with a clear description of the aim of this work (line 83ff) and begin the section “Materials and Methods” with a description of the data, process and requirement analysis.

In order to be able to insert the description of the software environment and the computational models and analytics from the end of the manuscript (in terms of comprehensibility and readability), we also had to move the section “Generic overall design and data flows” from the results to the methods section for better readability (line 166).

Results and some point for discussion

- Provide the link to the demonstrator earlier in the result section.

According to the suggestion, we added the link to the demo server and the walkthrough video at the end of the introduction (line 162ff).

- Have you considered to use syntaxtic data instead of pseudonimised data for the research application?

We assume that the reviewer means synthetic data and thank him/her for the thought-provoking question. We had not previously thought of the possibility of providing synthetic data and see this as an interesting application for the research view. Our framework could in principle do this by implementing a computer model in the Model and Simulation Server to artificially generate data (e.g., “virtual patient twins” or artificial training data for AI projects). We have added this interesting aspect to the discussion of the manuscript (line 431ff).

- In fig 2 it seems that data is used in the purper block at the bottom (to build models) is separated from the pseudonimisation while I believe that you use pseudonimised data for model development.

It is true that a particular computational model implemented in the Model and Simulation Server (the purpur block in Fig 2) receives data only from the Application Database, which itself contains only pseudonymized data as described in the legend of Fig. 2 and in the text (line 191ff). While Figure 2 only illustrates re-identification (III), Figure 1 also describes and illustrates pseudonymization.

As a result of the model execution (regardless of whether in a clinical or research context), a prediction or analysis for a particular (pseudonymized) patient is created and sent back to the Application Database. Thus, the Application Database contains only pseudonymized information, both data and model results/predictions. To make this even more clear for the reader, we have slightly changed the explanation in the text (line 187) and adapted Figure 1.

- Why is the pseudonimised data reidentified? I think you use pseudonimised data to develop a model, then apply this model on new cases in practice that can not be and donot need to be pseudonimised in the context of routine care. But the patients used to develop the model do not need to be deidentified, is not it?

The computational models and analyses used in both clinical care and scientific research are implemented into the Model and Simulation Server and receive (for data protection reasons) only pseudonymized patient data from the Application Database (cf. Fig1, Fig 2, line 191ff). The analyses and individual predictions resulting from the model executions are returned back to the Application Database and are available as pseudonymized data within the respective research or treatment context. In contrast to the research view, a re-identification of the patient data and prediction is required in the clinic view. This is done by requesting the Independent Trusted Third Party. As we mentioned in the previous comment, we have slightly changed the explanation in the text (line 187) and adapted Figure 1 to make this even more clear for the reader.

- What does the abbreviation gICS mean?

gICS is an acronym for **g**eneric **I**nformed **C**onsent **S**ervice. It is a name for a module of the MOSAIC Trusted Third Party tools that manages the consents. We have added the explanation of the acronym on first use (line 220ff). For completeness, we have also added the explanations of the acronyms E-PIX (line 219ff) and gPAS (line 221ff).

- You wrote “This ensures that only data from patients who gave their written consent to the respective use are finally provided” but patients do not have to give consent that their data is applied to a developed model in routine care is not this? Or is this legalisation specific in your country?

We thank the reviewer for drawing attention to this. It is true that a patient does not have to give consent for his or her data to be used in a developed model in routine care. But our goal was to provide a generic framework for in silico predictions for both clinical research and patient care. Therefore, we use the tools of the MOSAIC TTP server also for routine care to map specific clinical data to a specific treatment context using particular department-specific consent information within the TTP and apply appropriate pseudonyms. However, the department-specific “consent” is (in our case) merely a technical data object in the gICS module of the MOSAIC TTP server and is never signed by the patient. This generic data concept ensures that only clinicians have access to medical data and model predictions generated within their treatment context. For example, haematologists can only access data collected during haematology treatment, but not data collected during e.g., psychological treatment. In summary, with the consent data object of the TTP, we implement the legal basis for data usage. We clarified the concept of access control in the result section of our manuscript (line 304ff).

- How is uncertainty on the individual prediction modelled and visualised? Does the software solution provide any specific part to monitor the performance of the model and if needed recalibrate the model?

The reviewer raises an important point. Indeed, any model-based estimates should come along with an appropriate measure of uncertainty or confidence, respectively. However, the nature of this confidence region depends strongly on the underlying model, e.g., whether we use a statistical model for the prediction or whether one uses multiple realizations of a stochastic process. However, as a generic solution, we agree that our software solution needs to handle these additional features. While the particular realization for the data provision within the front-end is tailored for the problem in question, we use the example of recurrence predictions to illustrate how a confidence region can be estimated from the regression model and is provided along with the prediction. We adapted Figure 3 accordingly and also mention the model’s ability to integrate measures of uncertainty at several occasions throughout the manuscript (line 244ff, line 324ff).

With respect to the second question, we like to point out, that the underlying models (either statistical or dynamical models) are initially “trained” (i.e. estimation of model parameters) and then kept fixed for the integration in the software solution. For the example of the “model 1: Molecular monitoring during dose reduction in CML patients”, we apply a linear regression model and estimate the slope parameter for a particular patient, given the actual available BCR-ABL measurements. The decision, whether the particular outcome is critical or not, is based on a previously established result, which allows us to calculate a recurrence probability for any given (linear regression) slope parameter. In this respect, limitations of the model *performance* are reflected by insufficient model fits and larger uncertainties of the parameter estimates. We argue that a critical assessment of the general model suitability should always be in place by the user. Thus, whereas the generic framework allows to provision of certain models/model predictions, the decision which of the provided models should be included into the clinical decision is a clinical question. On the other hand, the generic framework also allows the inclusion of further models (algorithms), which might be developed on request by clinicians/users.

In order to bring this aspect to the readers’ attention, we briefly refer to it in the Discussion section (line 437).

- How is or can the demonstrator be integrated with the CIS/EHR?

We thank the reviewer for the question. The demonstrator is currently not integrated within a CIS/EHR, because the implementation of the integration depends on the respective use case and on the technical possibilities of the respective CIS/EHR. One possibility could be to extend our application with a RESTful API that would allow the CIS/EHR to query resources from our framework, such as the latest model prediction or analysis for a specific patient. In the case that the CIS/EHR system provides for such purpose a Restful API, this interface could also be used. Therefore, further projects will need to determine the specific requirements and select appropriate methods for implementing the integration.

For demonstration purposes of how a potential integration with a CIS/EHR might look like, we developed a REST API as a prototype to query all model predictions for a patient within a treatment context using the local CIS/EHR identifier. As a result, the CIS/EHR system receives a list of model predictions with metadata, including a link to a view of model results that can be accessed through or embedded in the system. For a description of the API and instructions for testing, see the documentation included in the repository or visit https://predict.imb.medizin.tu-dresden.de/docs/rest.html.

We have referred to these aspects in the results (line 343ff) and in the discussion section (line 436ff).

- The topic of data pseudonimisation got a lot of attention but the also mentioned data quality aspects are nearly described. What does this solution do in that regard?

The conceptual work on data integration in scientific research (that our software framework is based on) includes several methods to achieve integrated and curated data like data auditing, standardization, data linking, consolidation, data enhancement, data cleansing and verification. Data quality requires a continuous process and effort and can only be achieved through extensive standardization, centralization and automation of all data processing. Since data quality is an enormously high priority and data are cleansed before integration into the Clinical Data Repository (see Fig 1, ETL: Data Integration), we assume that the pseudonymized data provided by the (Research) Data Management (see Fig 1, ETL: Data Distribution) are quality assured. We included this assumption in the manuscript (line 120), and also addressed the data quality aspect in the discussion (line 402ff).

- The demonstrator presents results from the prediction model for some demo patients but it does not help the physician to understand how the model came to this prediction (which predictors contribute most to the outcome). This is known to be a very important feature to get models implemented and used in practice.

We thank the reviewer for the remark and we agree with it. However, the manuscript is primarily intended to describe a generic framework, assuming that users are familiar with the models/algorithms they apply (use within their decision-making). Still – as we clearly see the point raised by the reviewer - we have adapted the application so that a reference publication is provided for each model (this could also be the reference to a preprint or website). We have amended Fig 3, its legend (line 338) and the manuscript (line 327ff, L436ff).

Comments from Reviewer 2:

The manuscript deals with an important question within the medical informatics community: providing useful recommendations to clinicians and researchers re-using clinical data, including data privacy protection technologies. The proposed framework is successfully tested and the results are reported in a visual and clear way.

Major revision

In my opinion, the manuscript could address the research question in a more innovative manner, as the previous work (Reference n.15) already successfully demonstrates the integration of model results in clinical practice and suggests a framework for it. This new version of the framework includes new technologies and interesting innovations such as the TTP and the inclusion of the MOSAIC Tools, as well as updated models and dashboard. Prior publication, I would suggest a more thorough comparison of the two frameworks and an evaluation of the current one in terms of efficiency and satisfaction.

According to the journal guidelines, the source code of the system and the models must be published.

We thank the reviewer for the valuable comments. In the discussion, we now compared both frameworks more thoroughly, such that we more clearly highlight the innovative aspect of this work (line 395ff).

Our main goal was to develop a *prototype* that *exemplifies* how individual models or algorithms (including analyses results and/or predictions) could be implemented in clinical practice with all the requirements, mentioned in the manuscript. We agree that the evaluation in terms of usability, satisfaction, and effectiveness using a concrete application should be the subject of further projects. Therefore, appropriate medical needs and concepts must be defined together with the treating physicians and scientific researchers, and specific models and analysis pipelines must be developed and validated. Depending on the intended purpose of the application, compliance with regulations such as the European Medical Device Regulation (MDR) or the In-vitro Diagnostics Regulation (IVDR) must also be ensured. Furthermore, for implementation in the clinical routine workflow, the required data flow processes for managing identifying data via the Independent Trusted Third Party and for providing the integrated and cleansed data via the (Research) Data Management must be developed, implemented, and monitored. These extensive tasks went beyond the scope of our conceptual work and will need to be the focus of future projects.

We briefly described these tasks and included the need for an evaluation of effectiveness and satisfaction that can be done through usability studies in the discussion section (line 438ff).

We apologize for missing to provide the link to the source code. The source code of the latest server application can be downloaded from the GitLab repository at https://gitlab.com/imb-dev/predictdemo. Furthermore, the source code archived at the time of publication can be found at https://zenodo.org/record/7655167#.Y_Kdiy1XaqA. This repository also includes the computational models and test datasets implemented in the demo server as well as the developer documentation, including initial installation instructions. We have added this information to the result section (line 293ff).

Minor revision

L37„and a research perspective focusing on the exploration of aggregated, but pseudonymized data." Aggregated but pseudonymized is contradicting.

We agree. By this, we mean that the researcher is provided with pseudonymized data that can be used for analysis. We have deleted the phrase "aggregated, but" to avoid confusion (line 37)

L76 „we need to ask how health data“

I would rephrase this.

We rephrased it to “the question arises how health data” (line 77).

L113 „loading into a data warehouse“

„Data warehouse“ does not represent all of the end targets of an ETL process.

We agree, that an end target of an ETL process can be not only a Data Warehouse, but also other data systems, such as our framework (cf. Fig 1, ETL:Data Distribution). We rephrased “This procedure is often denoted as ETL (Extract-Transform-Load) process.” to “Such data processing is often denoted as ETL process.” (line 119)

We also changed the term “Data Warehouse” to “Clinical Data Repository (CDR)” throughout, as it is more appropriate according to Gartner’s definition, see https://www.gartner.com/en/information-technology/glossary/cdr-clinical-data-repository.

L117 „This ensures that the pseudonymized medical data do not allow any conclusions about a patient's identity. “

This statement could be refuted.

We agree that in the case of human genetic data and also of data on rare diseases, conclusions about a patient’s identity are not completely impossible. We have added this aspect in the manuscript (line 125ff).

L135 „data protection laws“

What laws exactly?

Our solution is compliant with the (European) General Data Protection Regulation (Datenschutzgrundverordnung DSGVO/GDPR), the Germany Federal Data Protection Law (Bundesdatenschutzgesetz BDSG), the State Data Protection Law of Saxony and the State Hospital Act of Saxony. At the national level in particular, legal principles such as data avoidance and frugality [§3a of the German Federal Data Protection Act (Bundesdatenschutzgesetz, BDSG)] and the requirement to separate identifying data from other personal data (§40 of the BDSG) have been observed. Since no state-specific laws were applied that would currently preclude the use of the framework in other regions of Germany, only the DSGVO/GDPR and the Germany Federal Data Protection Law were added to the manuscript (line 147ff).

L156 „standards for data security and pseudonymisation“

What standards?

We followed the recommendations of the *Data Protection Working Group of the technology and method platform TMF e.V.* for a regulation-compliant pseudonymization service that fully meets the requirements of data protection.

(https://www.tmf-ev.de/EnglishSite/WorkingGroups/Dataprotectionworkinggroup.aspx). The TMF provides a guideline proposing an Independent Trusted Third Party to address typical challenges in data protection and ethics. We have clarified our statement in the manuscript (line 171ff).

Comments from Reviewer 3:

Hoffmann et al. describe a relatively comprehensive and practical proposal for both sharing pseudonymized patient record data derived from the EHR systems for predictive modelling, and returning the model data via re-identification for individual patient use.

The manuscript text is well written, concise, and sufficient in detail. Figures would benefit from editing by a medical graphics designer/artist.

We thank the reviewer for the positive feedback and the suggestions for improvement. We also see potential for optimization in the figures and improved them accordingly.

Specific questions

1. Is the proposed system compatible with common cloud infrastructures (e.g. Microsoft Azure) used for EHRs for most hospitals.

The migration of our software solution to a cloud infrastructure, such as Microsoft Azure, is - from the technical point of view - possible. However, the data protection requirements for the respective use case must be taken into account. As this implies also country-specific issues (e.g. rather strong rule in Germany), we intentionally avoided to mention cloud solutions in the description of our generic framework.

2. Has the solution been installed and validated in production use in a real hospital environment? Any experience on e.g. Epic integration?

So far, we have not implemented the software framework in a real hospital environment. The main goal of this work was the development of a *prototype* that *exemplifies* how individual model predictions and analyses could be implemented in clinical practice with all the requirements, mentioned in the manuscript. The development and implementation of particular applications in a concrete hospital environment are beyond the scope of our conceptual work due to the extensive tasks involved (see comments to reviewer 1 and reviewer 2) and must be carried out in independent projects. We have clarified the goal of this work in the introduction section (line 83ff) and amended some future directions in the discussion section of the manuscript (line 435ff).

For this reason, we do not yet have experience integrating with a CIS/EHR (e.g., EPIC), but are aware that this is necessary for a usable clinical decision support system. Although the specific implementation of the interface depends on the particular CIS/EHR and use case, we have developed a REST API prototype that would allow the CIS/EHR to query all model predictions for a patient in their specific clinical care context and provide the model results embedded in or through the system. We have amended this aspect in the abstract (line 42ff), results (line 343ff), and discussion (line 436ff).

3. The authors describe the solution as “generic”, yet the use cases presented are the same from their previous work – CML outcome modeling. As CML is an exceptionally “simple” cancer with only one marker (blood BCR::ABL1-transcript level) sufficient for patient follow-up. How does the “clinical view” handle longitudinal visualization of more complex diseases, like breast cancer, with multiple markers (e.g. ctDNA) and other outcome measures (radiology, pathology) routinely used for follow-up and outcome modeling?

We thank the reviewer for the remark. Our solution is indeed generic in terms of the processes for handling/providing data, model predictions and analyses to support clinical decision-making in patient care and for scientific research purposes, because the software architecture is based on fundamental concepts. The models (or algorithms in general) as well as the presentation of the model results are specific, i.e. developed according to the requirements arising from the

individual use case. Here – the reviewer is completely correct - we use disease (CML)-specific *examples*, which have already been validated and also published. The software solution describe in the manuscript at hand, is to be understood as a technological basis to integrate such individually developed models, computer simulations or data analysis algorithms into the clinical data and information processes. We have adapted the manuscript to make this distinction more explicit, i.e. to make clear that the currently implemented models and the presentation of their results are examples (use cases) only, but that other models/algorithms could immediately be integrated into the described framework, i.e. that the framework itself is not disease- or model- specific at all (line 428ff).

4. Would be good to mention that implementation of a common data model for the clinical data (e.g. FHIR, OMOP) would ensure scalability of the solution to other environments and data owners

We thank the reviewer for the comment and agree with it. We added this note in the discussion section (line 448ff).

5. Can the solution utilize data from multiple data owners in a federated way (no central data repository; no transfer of primary patient data)?

Theoretically, it is possible to add such a layer around our presented software framework. However, currently, there is no infrastructure in place that would address its use for federated learning approaches in the context of our described framework. The need for that certainly would depend on the particular model in question. Please note that the scope of this paper is not concerned with the wide scientific field that is „federated learning“, but primarily focuses on the development of a flexible and easy-to-implement simulating environment in a specific hospital setting. Although we definitely agree that the inclusion of federated analysis might be very helpful and would broaden the applications, the deployment in a federated learning environment will require further research as well as expertise in technology security and legal requirements. We consider this as important, but see it as future work. We thankfully acknowledge the overlap provided by the reviewer and included a respective comment in the discussion section of the manuscript (line 458ff).

6. Will the solution likely be EHDS-compliant?

We are aware of the legislative proposal for the EU regulation and see enormous potential in it. However, as this first draft of legislation does not yet give any concrete indication of which technologies and standards will be used and what exactly will be built, we currently do not see any dedicated reason to evaluate our software framework in this respect. Nevertheless, we are convinced that our solution is suitable for the integration of new emerging technologies and we will continue to follow the developments towards a European Health Data Space with interest. We referenced this in the discussion section (line 448ff).
